# Initial evidence of reduction of malaria cases and deaths in Rwanda and Ethiopia due to rapid scale-up of malaria prevention and treatment

**DOI:** 10.1186/1475-2875-8-14

**Published:** 2009-01-14

**Authors:** Mac Otten, Maru Aregawi, Wilson Were, Corine Karema, Ambachew Medin, Worku Bekele, Daddi Jima, Khoti Gausi, Ryuichi Komatsu, Eline Korenromp, Daniel Low-Beer, Mark Grabowsky

**Affiliations:** 1 World Health Organization, Global Malaria Program, Geneva, Switzerland; 2 Ministry of Health, Kigale, Rwanda; 3 World Health Organization, Addis Ababa, Ethiopia; 4 Ministry of Health, Addis Ababa, Ethiopia; 5 World Health Organization, Harare, Zimbabwe; 6 Global Fund for AIDS, TB and Malaria, Geneva, Switzerland

## Abstract

**Background:**

An increasing number of malaria-endemic African countries are rapidly scaling up malaria prevention and treatment. To have an initial estimate of the impact of these efforts, time trends in health facility records were evaluated in selected districts in Ethiopia and Rwanda, where long-lasting insecticidal nets (LLIN) and artemisinin-based combination therapy (ACT) had been distributed nationwide by 2007.

**Methods:**

In Ethiopia, a stratified convenience sample covered four major regions where (moderately) endemic malaria occurs. In Rwanda, two districts were sampled in all five provinces, with one rural health centre and one rural hospital selected in each district. The main impact indicator was percentage change in number of in-patient malaria cases and deaths in children < 5 years old prior to (2001–2005/6) and after (2007) nationwide implementation of LLIN and ACT.

**Results:**

In-patient malaria cases and deaths in children < 5 years old in Rwanda fell by 55% and 67%, respectively, and in Ethiopia by 73% and 62%. Over this same time period, non-malaria cases and deaths generally remained stable or increased.

**Conclusion:**

Initial evidence indicated that the combination of mass distribution of LLIN to all children < 5 years or all households and nationwide distribution of ACT in the public sector was associated with substantial declines of in-patient malaria cases and deaths in Rwanda and Ethiopia. Clinic-based data was a useful tool for local monitoring of the impact of malaria programmes.

## Background

In 2005, the global malaria community committed itself to the goal of reducing the global malaria burden by at least 50% by 2010 [1]. The recommended method to achieve this target is > 80% coverage of the four main malaria control tools: long-lasting insecticide treated bed nets (LLIN), indoor residual spraying (IRS), intermittent presumptive treatment of pregnant women (IPT), and treatment with effective medicines, principally artemisinin-based combination therapy (ACT).

Starting from a low baseline in 2005 [2], countries are now engaged in efforts at rapid scale-up to reach the 2010 coverage targets. This scale-up to achieve disease-control targets raises several questions. What is the causal relationship between the coverage targets and the disease control goals: does 50% disease reduction require 80% coverage of all interventions or can partial coverage of selected interventions more efficiently achieve the goal ? How rapid is the impact seen after scale-up ? How can impact be measured – can one use existing data in the public health system or are special surveys required ? To address these questions, the impact of malaria control on health facility burdens was assessed in selected areas of Rwanda and Ethiopia, two countries that had recently conducted national scale-up of the distribution of LLIN and use of ACT.

## Methods

Data were collected during two weeks in November–December 2007 by Ministry of Health and WHO personnel.

### Interventions

In Rwanda, the Ministry of Health (MOH) introduced LLIN and ACT nationwide within a two-month period, September to October 2006. In September 2006, the MOH conducted a mass distribution of 1.96 million LLIN to children < 5 years, integrated with measles vaccination. (In comparison, Rwanda's population was around 9.5 million in 2006.) During a household survey 8 months after this campaign, LLIN use in children < 5 years old was 60% (unpublished MOH Malaria Indicator Survey, 2007). ACT was introduced nationwide quickly in October 2006 to public-sector health facilities throughout the country.

In Ethiopia, the MOH conducted continuous mass distribution of LLIN from September 2005 to December 2007, aiming to distribute one LLIN per two persons at risk. In 2005–2006, 16.3 million LLIN were received, and all except 2.2 million were distributed nationwide by end of 2007 [3]. ACT were first distributed in the public sector in 2005. Over 2005–6, 10.2 million ACT courses were distributed. In comparison, the national population of Ethiopia was 81 million in 2006, out of which 55 million people were living in areas with (stable or unstable) malaria transmission. Results from a Malaria Indicator Survey conducted in October to December 2007 indicated that 65% of households in areas < 2000 meters elevation had at least one LLIN [3].

### Selection of districts

To evaluate intervention effect on malaria morbidity and mortality burden in both Ethiopia and Rwanda, districts were selected with two main objectives: areas with stable *Plasmodium falciparum *malaria transmission and achievement of wide geographical representation.

In Ethiopia, two districts were conveniently selected from each of four major Regions than have areas with moderate malaria: Oromiya; Southern Nations, Nationalities, and Peoples (SNNP); Amhara; and Tigray.

In Rwanda, two districts in all five provinces were sampled, hence covering 10 of 33 districts.

### Selection of health facilities

**Figure 1:**
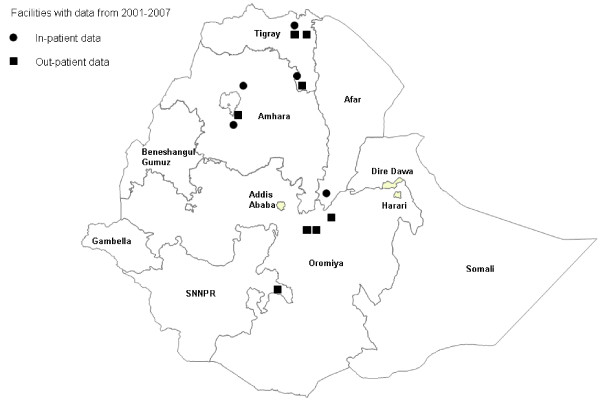
**Location of hospitals and health centers that were selected for data collection and had complete data for 2001–2007, November 2007, Ethiopia**.

**Figure 2:**
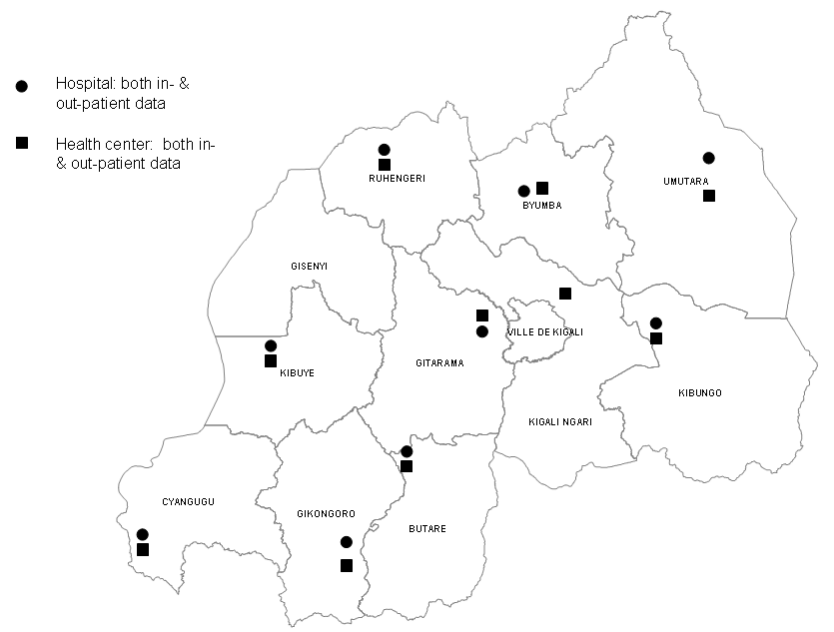
**Location of hospitals and health centers that were selected for data collection and had complete data, December 2007, Rwanda**.

Figures 1 and 2 show the location of selected facilities with complete data in both countries. Per selected district, one hospital and one out-patient health center were sampled. In Ethiopia, facilities with complete data were spread over approximately half the country (Figure 1). In Rwanda, facilities with complete data were spread throughout the country (Figure 2).

In Rwanda, one health center was excluded from analysis because of incomplete data, leaving nine out of the total national 39 hospitals, and 10 out of the 439 national health centers in the sample. All sampled facilities performed malaria smears on all suspected malaria cases, and the out-patient cases that were analysed represent exclusively laboratory-confirmed cases.

In Ethiopia, health facilities that did not have data going back to 2001 were excluded. Some health facilities had complete data for some age groups (for example, total for all ages, but not < 5 years). Five health facilities had complete in-patient data for both cases and deaths for all ages (total), four facilities had in-patient case data by age group (< 5 and ≥ 5 years), and three facilities had in-patient death data by age group. Complete out-patient data was available from eight facilities for all ages combined, and from seven facilities by age group. For Ethiopia, the proportion of out-patient cases that had laboratory examinations was not recorded.

### Data and indicators analysed

Interviewers abstracted data either from health-facility copies of national surveillance forms, other health information forms, or from patient registers. At least two persons visited each district for at least two days. Monthly data between January 2001 and November 2007 were abstracted. From hospitals, data on in-patient malaria and all-cause cases and deaths, and from out-patient health centers and hospital out-patient departments, data on laboratory-confirmed out-patient malaria cases and all-cause attendances were collected. For all indicators, data were stratified in two age groups, < 5 years and ≥ 5 years, as far as available.

For facilities which had one or two months of data missing in a year, data were imputed as the average of the month prior and month after the month(s) of missing data. In Ethiopia, in-patient data was imputed for 15 of 586 health-facility-months. In Rwanda, out-patient data were imputed for 55 of 1,595 health-facility-months and in-patient data for 1 of 133 facility-years.

### Evaluation of time trends and intervention impact

To assess changes in indicators associated with the scale-up of LLIN and ACT, numbers of malaria and non-malaria cases and deaths were compared between years before LLIN and ACT introduction, and the year thereafter.

Analyses were limited for all years to January to October because October 2007 was the last month with complete data in both countries. In Ethiopia, 2001–2005 as the reference period was used because most LLIN and ACT were distributed in September 2005 or later, so these interventions would not have much effect on data from January to October 2005. The years 2001–2006 were used as the reference period for Rwanda, where interventions were scaled-up in September–October 2006.

Two methods were used to estimate the decline in malaria cases and deaths after intervention. First, 2007 data were compared with the average of the pre-intervention period (2001–2006 for Rwanda and 2001–2005 for Ethiopia; Table 1, column 5 for malaria and column 9 for non-malaria).

**Table 1:** Percentage change in malaria and non-malaria cases and deaths in 2007 compared to pre-intervention reference period, in selected health facilities, in persons < 5 years and ≥ 5 years, Ethiopia and Rwanda, from January to October each year. Positive percentage indicates decline, negative percentage indicates an increase.

		**MALARIA**				**NON-MALARIA**			
		
Indicator	Age (years)	Annual average, reference period	2007	Decline in 2007		Annual average, reference period	2007	Decline in 2007	
				Observed, compared to average over 2001–5/6*	Adjusted for linear trend over 2001–5/6† (95% CI)			Observed, compared to average over 2001–5/6*	Adjusted for linear trend over 2001–5/6† (95% CI)
**Rwanda**									
In-patient cases	< 5	7,686	3,473	55%	64% (60–68)‡	6,043	7,793	-29%	9% (-7 – 20)
	5+	10,250	4,403	57%	59% (53–64)‡	27,384	45,275	-65%	-7% (-20 – -3)
	All	17,936	7,876	56%	61% (57–65)‡	33,427	53,068	-59%	-5% (-17 – -6)
In-patient deaths	< 5	153	51	67%	68% (56–75)‡	221	259	-17%	4% (-20 – 21)
	5+	207	187	10%	8% (-32 – 29)	644	939	-46%	-10%(-30 – -6)
	All	360	238	34%	34% (9–48)‡	865	1,198	-39%	-6% (-27 – -9)
Laboratory-confirmed out-patient cases	< 5	11,216	4,665	58%	63% (55–68)‡	38,996	61,108	-57%	4% (-7 – 13)
	5+	19,221	9,254	52%	54% (39–62)‡	120,587	210,644	-75%	3% (-5 – 10)
	All	30,437	13,919	54%	57% (46–64)‡	159,583	271,752	-70%	4% (-5 – 11)
**Ethiopia**									
In-patient cases	< 5	866	232	73%	83% (79–86)‡	1,140	1,245	-9%	25% (20 – 30)‡
	5+	3,381	1,093	68%	69% (6–82)‡	5,993	7,968	-33%	11% (4 – 16)‡
	All	4,540	1,357	70%	75% (51–84)‡	7,263	9,391	-29%	14% (8 – 19)
In-patient deaths	< 5	11	4	62%	*Too few data*	23	20	13%	*Too few data*
	5+	28	7	75%	3% (-103 – 54%)	65	45	31%	-117% (-150 – -71)
	All	135	28	79%	84% (67–89)‡	294	408	-39%	-10% (-24 – -2)
Laboratory-confirmed out-patient cases	< 5	5,495	801	85%	69% (45–83%)‡	30,228	43,688	-45%	-15% (-30 – -3)‡
	5+	17,903	2,841	84%	91% (87–93)‡	160,339	162,565	-1%	20% (12 – 26)‡
	All	31,493	6,131	81%	85% (77–89)‡	184,777	209,244	-13%	4% (-7 – 12)

* Reference period was 2001–2006 for Rwanda and 2001–2005 for Ethiopia

† Percentage change was calculated by comparing reported number of cases/deaths in 2007 with predicted number of cases/deaths in 2007 based on linear trend of 2001 to 2005/6.

‡ Significant difference. A 95% CI not including the 0% value indicates that the difference in 2007 compared to the pre-intervention period was statistically significant (p < 0.025).

The second method accounted for possible time trends in indicators that started before the interventions and would thus be unrelated to the interventions – such as population growth, improved health facility access and attendance. Here, the observed 2007 value for each indicator was compared with its corresponding, expected value for that year based on the linear trend over 2001 through 2005/6 (using *SPSS Inc*., version 14.0 for linear regressions and 2-tailed Student's T-tests for assessing statistical significance of the difference between observation and expectation). In this second analysis, any decreases in malaria indicators observed in 2007 which could be predicted simply from a trend of decline in that indicator over preceding years were thus not attributed to the interventions, whereas decreases larger than those apparent over previous years and decreases that started in 2007 were attributed to the interventions.

For Rwanda, in addition, the month-to-month trend in malaria slide positivity rate was analysed (including all 12 months of all calendar years), alongside the month-to-month trends in out-patient malaria laboratory-confirmed cases, in-patient malaria cases, and non-malaria inpatient cases.

## Results

### Time patterns of intervention scale-up and start of health facility impact

**Figure 3:**
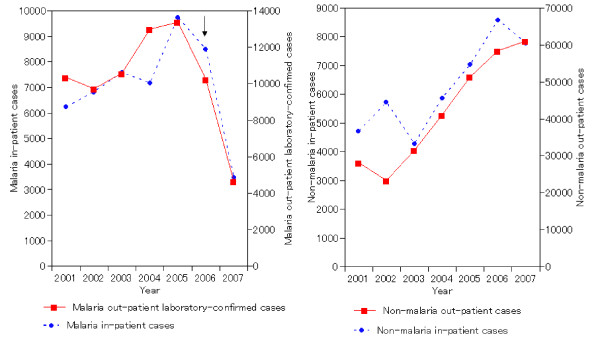
**Malaria and non-malaria in- and out-patient cases, children < 5 years old, January to October 2001–2007, Rwanda**. LLIN = long-lasting insecticidal nets, ACT = artemisinin-based combination therapy medicines.

**Figure 4:**
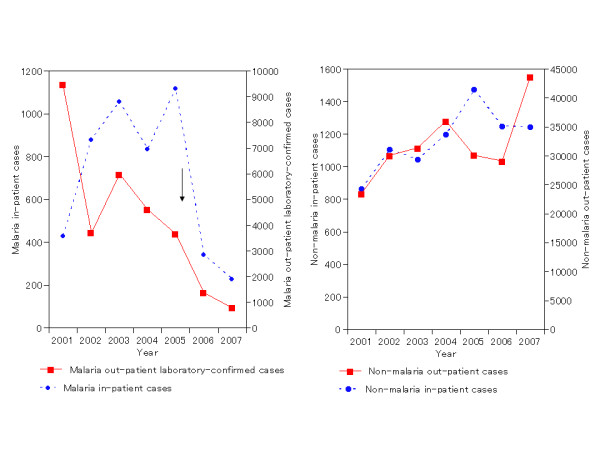
**Malaria and non-malaria in- and out-patient cases, children < 5 years old, January to October 2001–2007, Ethiopia**. LLIN = long-lasting insecticidal nets, ACT = artemisinin-based combination therapy medicines.

Figures 3 and 4 show the time trends in in-patient malaria and non-malaria cases in children < 5 years by calendar year. For both countries, declines in malaria indicators were apparent that started within a year after scale-up of LLIN and ACT.

In Rwanda (Figure 3), the decline observed in 2007 was very sharp and distinct, following a trend of moderate increase in malaria cases over 2001–5. Non-malaria cases increased throughout 2001 to 2007, possibly indicating a general trend of improving health facility access and attendance – not related to malaria intervention – over this period.

Data for Ethiopia (Figure 4) also showed a marked decline in malaria cases in 2007 (possibly starting in 2006). Here, however, the causal relationship with the scale-up of LLIN and ACT was less obvious than in Rwanda, because of marked fluctuation in malaria cases over the pre-intervention period 2001–2005. Non-malaria cases in Ethiopia also fluctuated considerably from year to year, with perhaps a slight overall increase over 2001–2007.

### Rwanda

Comparing 2007 against the average of 2001–2006, observed declines in the two age groups ranged from 52% to 67% among the three malaria indicators, except for in-patient malaria deaths in those ≥ 5 years which declined by only 10% (Table 1, column 5). Declines in children < 5 years old were 55% for in-patient malaria cases, 67% for in-patient malaria deaths, and 58% for out-patient laboratory-confirmed cases.

Adjusting for pre-intervention trends, the estimated declines in the two age groups ranged from 54% to 68% for the three indicators, and these were all highly significant (Table 1, column 6). An exception was in-patient malaria deaths in those ≥ 5 years, for which the decline was estimated as a non-significant 8%. Since most malaria indicators had slightly increased between 2001 and 2006, this adjustment slightly increased the estimated impact in children < 5 years old, to declines of 64% for in-patient malaria cases, 68% for in-patient malaria deaths, and 63% for out-patient laboratory confirmed cases.

Non-malaria out-patient cases and in-patient cases and deaths, in contrast, were all higher (by 17% to 75%) in 2007 compared to the average over 2001–2006 (Table 1, column 9). For none of the non-malaria indicators was the observed value in 2007 statistically different from that expected based on the trend of increase over 2001–2006 (Table 1, column 10).

**Figure 5:**
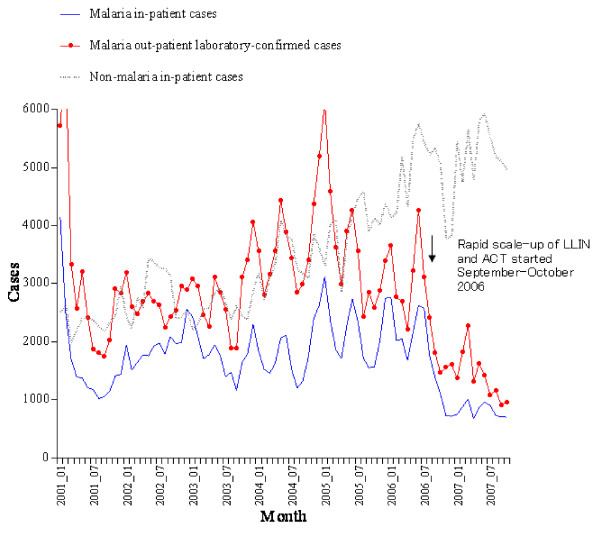
**In-patient malaria cases, out-patient laboratory-confirmed cases, and in-patient non-malaria cases, by month, all ages, January 2001 to October 2007, Rwanda**. LLIN = long-lasting insecticidal nets, ACT = artemisinin-based combination therapy medicines.

Rwanda was unique because nationwide distribution of LLIN and ACT occurred within only 60 days in September–October 2006. As an obvious effect, the monthly number of malaria cases showed a sharp immediate decline in November and December of that year (Figure 5).

As an additional analysis adjusting for month-to-month fluctuations, the malaria slide positivity rate, in-patient malaria cases and out-patient malaria laboratory-confirmed cases all showed highly significant reductions (p < 0.001) in 2007 compared to 2001–6. In contrast, the number of non-malaria in-patient cases was not significantly different in 2007 compared to 2001–6 (p = 0.74), when adjusting for month-to-month fluctuations.

### Ethiopia

Comparing 2007 against the average of 2001–2006, observed declines ranged from 62% to 85% among the three malaria indicators in the two age groups (Table 1, column 5). Observed declines in children < 5 years old were 73% for in-patient malaria cases, 62% for in-patient malaria deaths, and 85% for out-patient laboratory-confirmed cases.

Adjusting for pre-intervention trends, the estimated declines in the two age groups ranged from 3% to 91% across the eight malaria indicators and age groups for which statistical testing was possible, with seven out of eight effects being statistically significant (Table 1, column 6). For in-patient deaths in children under five years of age, too few data points were available to allow statistical testing.

In comparison, non-malaria out-patient cases and in-patient cases and deaths were higher (by 1% to 45%) in 2007 compared to the average of 2001–2006, except for in-patient deaths which declined by 13% for those <5 years and 31% for those ≥ 5 years (Table, column 9). After adjustment for trends over 2001–2006, non-malaria in-patient cases declined significantly by 11–25%, while outpatient cases increased in children under-five, but decreased in the age group over five years (Table 1, column 10). For in-patient deaths, no significant changes were apparent in 2007, when adjusting for prior time trends.

## Discussion

This study documents for the first time marked reductions in malaria cases and deaths in health facilities in two medium- and large-sized countries following large-scale distribution of LLIN and ACT. In particular in children under-five, declines of malaria cases and deaths were dramatic (~50% or higher), and occurred within one year of the scale-up of malaria interventions. This suggests that mass distribution of LLIN and nationwide roll-out of ACT conducted within months of the start of an expected malaria season may reduce cases and deaths during that transmission season.

In both Rwanda and Ethiopia, declines were of similar magnitude (~50% or higher) for in-patient cases and deaths, and out-patient laboratory-confirmed malaria cases. In Rwanda, all suspected malaria cases had laboratory examination. Since laboratory-confirmed diagnosis has better specificity for malaria than clinical diagnosis [4], this similarity supports the interpretation that apparent impacts are real, rather than being artifacts of changing patterns of diagnostic practices in the sampled facilities.

In Rwanda, the decline of in-patient and out-patient laboratory-confirmed malaria cases occurred in the face of increases in in-patient and out-patient non-malaria cases over 2001–2005, which probably reflected the introduction of health insurance schemes, resolution of civil conflict, and improvement of health services. The exceptionally low percentage decline in in-patient malaria deaths in Rwandans ≥ 5 years old was due to the doubling of reported malaria deaths in a single hospital (Nyanza) in the year 2007 (51 deaths, compared to < 25 annual malaria deaths during 2001–2006). As also non-malaria deaths in Nyanza hospital increased by 54% in 2007 compared to 2001–5, this sudden increase may relate to non-malaria factors. In any case, numbers of malaria deaths analysed (in both Rwanda and Ethiopia) were small, resulting in wide uncertainty ranges around mortality impact estimates.

In Ethiopia the strength of evidence was limited, first, by the small number of facilities with complete data for both age groups starting in 2001. Whereas the original sample had covered 13 out-patient and seven in-patient health facilities, only eight out-patient and five in-patient facilities had complete data over the period 2001–2007 (for at least all-ages combined) allowing inclusion in the present analysis. More fundamentally, the unstable nature of malaria transmission in Ethiopia [5] and the large year-to-year fluctuations in health facility burdens, also for non-malaria cases and deaths (Figure 4), make it impossible to draw firm conclusions yet regarding the causal relationship between the observed malaria declines and LLIN and ACT scale-up. Of note, Ethiopia experienced marked epidemics in 2003–4 [6,7], after which declines in malaria indicators were expected even in the absence of intervention impact. Nevertheless, a repeat of the current analysis with a few additional years of post-intervention data could be expected to yield conclusive evidence as to the impact of the national LLIN and ACT scale-up.

The magnitudes of declines found in malaria indicators are in line with those reported from similar studies in small-scale areas of Kenya and Zanzibar. In three hospitals along the Kenya coast, in-patient pediatric malaria cases declined 28–63% as of March 2007, after distributions of ITNs and ACT [8]. In District A in Zanzibar, in-patient malaria cases and deaths in children declined by 77% and 75%, respectively, within 24 months after the introduction of ACT in all 13 health facilities (and prior to substantial distribution of LLIN) [9].

The limitations were the following. Districts and health facilities were not randomly selected, but constituted a (stratified) convenience sample, selecting those sites where intervention scale-up had been relatively rapid and successful and where health facility data were of relatively good quality (e.g., in Ethiopia, excluding facilities where records did not go back to 2001). Therefore, estimated impacts cannot be extrapolated to the countries nation-wide. Also, while these results illustrate the benefits of rapid scale-up in the populations sampled, it would be inappropriate to extrapolate these findings to other countries with more intense malaria transmission, where interventions at similar coverage levels may have lower impact.

A more general limitation of health facility data is that they cover only the cases and deaths of patients who accessed the (public) health care system. Especially for ACT, it is possible that their coverage and impact is largely limited to the catchment populations of the facilities providing these drugs – with population-level impact diminishing by distance from health facilities. For this reason, it is difficult to extrapolate the observed health facility impacts to effects for the full populations living in the districts sampled. Inferences about population-level disease impact will typically require triangulation of several sources of data, including data from household surveys [10].

Third, in Ethiopia, indoor residual spraying (IRS) has been a well-established vector control intervention for a long period. It is applied in a focalized manner by targeting villages at risk for malaria epidemics. All districts visited had been applying IRS in a limited way during much of 2001–2007. The contribution of IRS to declines could not be estimated since IRS activities occurred throughout both pre- and post-intervention period.

Evaluation of impact is an essential part of modern programme management practice and will be needed by all high-burden African countries to meet the Roll Back Malaria goal of > 50% reduction in malaria-related mortality by 2010. Health facility data are important for quickly and continuously monitoring interventions with high impact at the health facility and district level. Facility-based surveillance may become even more important in the future, once wide-scale use of LLIN and ACT may change the epidemiology of malaria from stable endemic to unstable/epidemic. Areas of unstable and highly seasonal malaria will need to rely on continuous, timely surveillance to detect and respond to epidemics.

In conclusion, these initial data suggest that widespread distribution of LLIN and use of ACT in the public sector can result in marked reductions in the burden of non-severe and severe malaria morbidity and mortality seen in public-sector health facilities. International partners should urgently collaborate with national governments to ensure that all at-risk persons have access to appropriate vector control, including LLIN, and treatment with ACT. The magnitude of declines (≥ 50%) found in the studied facilities of Rwanda and Ethiopia was similar to that needed – at a population level – to reach the RBM target of 50% mortality reduction by 2010. It appears that marked reduction in malaria mortality can be achieved quickly and detected within a short time of scaling-up interventions, which may enable many African countries to make rapid progress towards the child survival targets in the Millennium Development Goals.

## Competing interests

The authors declare that they have no competing interests.

## Authors' contributions

MO led the conceptual design and writing; MA led field work and participating in writing; WW participated in field work and writing; CK and KG led the field work in Rwanda and commented on manuscript; AM organized field work and help with writing; DJ helped with the field work in Ethiopia; WK participated in data collection and reviewing the manuscript; RK contributed to the design and writing; EK contributed to the design, analysis, and writing; DLB contributed to the design and writing; and MG contributed to the design, analysis, and writing.
